# Seeds of Toxicity? Erythrocytes and Lead-Associated Kidney Damage

**DOI:** 10.1289/ehp.123-A42

**Published:** 2015-02-01

**Authors:** Julia R. Barrett

**Affiliations:** Julia R. Barrett, MS, ELS, a Madison, WI–based science writer and editor, has written for *EHP* since 1996. She is a member of the National Association of Science Writers and the Board of Editors in the Life Sciences.

Environmental lead contamination lingers despite decades of sharply declining use and release of the metal. As a consequence, low-level lead exposure remains common, with an average adult blood lead level in the general U.S. population of 1–2 μg/dL.^1^^,^^2^ Lead affects numerous organ systems, but specific mechanisms of damage are not always known. The authors of a new study in *EHP* present a hypothesis to explain lead-related toxicity in the kidney and support it with detailed *in vivo* and *in vitro* data.^1^

Current evidence suggests that kidney damage can occur at blood lead levels as low as 5 μg/dL.^2^ Specific populations, including people with preexisting kidney disease, diabetes, or hypertension, may be at even greater risk of effects of low-level lead exposure.^2^^,^^3^ Both *in vivo* and *in vitro* data highlight oxidative stress as a factor in lead-associated kidney damage, but it has been unclear how the stress is generated.^1^^,^^2^

**Figure:**
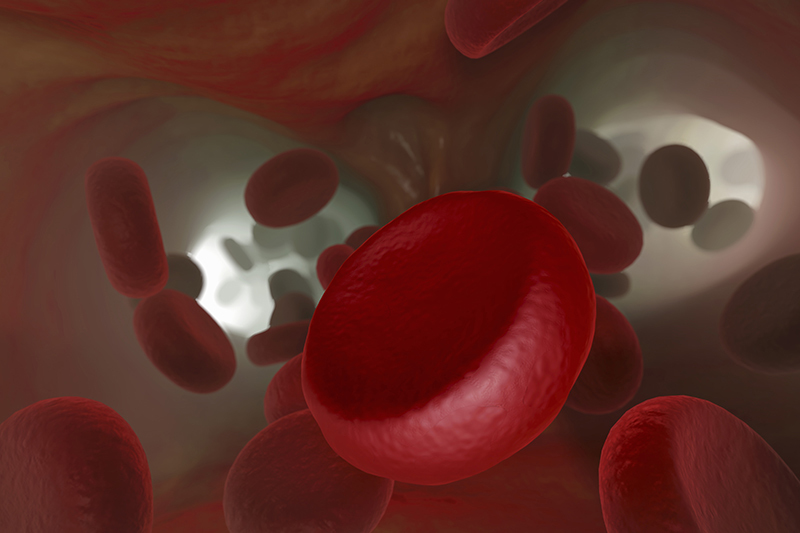
A new study explores the role that aging red blood cells may play in lead-related kidney toxicity. © Science Picture Company/Science Source

In the current study, the researchers wove observations from previous investigations into a testable hypothesis. They took into account deposition of iron—presumably from iron-rich red blood cells (erythrocytes)—in kidneys of individuals with renal disorders. They also considered the kidney’s role in clearing erythrocytes from circulation as the cells become old or damaged.

During a process called erythrophagocytosis, aging erythrocytes are enveloped and broken down by other cells. Erythrophagocytosis occurs primarily in cells of the spleen and liver, but proximal tubular epithelial cells in the kidney also have this capability. The signal that an erythrocyte needs to be removed from circulation comes from a compound called phosphatidylserine (PS). In a normal, healthy erythrocyte, PS is an internal cellular component, with no direct contact with the cell’s outer environment. Aged and damaged red blood cells begin to shift PS to the outer surface. Members of this research team previously found that lead exposure was associated with a surge in PS externalization, followed by enhanced erythrophagocytosis in the spleen.^4^

In this study the researchers hypothesized that lead exposure increases the number of PS-tagged erythrocytes, which are then consumed by the proximal tubular epithelial cells in the kidney. They further hypothesized that iron from the erythrocytes accumulates in the kidney cells, where it triggers the formation of reactive oxygen species, the first step in oxidative damage.

The ultimate biological outcome of toxic exposures is frequently a combination of two factors—individual responses of multiple tissues and, subsequently, complex interactions of altered tissues—says study coauthor Jin-Ho Chung, a professor in the College of Pharmacy at Seoul National University. “We considered that lead-associated nephrotoxicity must be interpreted in the context of systems biology, rather than as sole and isolated damage to the kidney,” Chung says.

Chung and his colleagues conducted a series of *in vitro* experiments with HK-2 cells^5^ and erythrocytes derived from volunteers’ blood samples. They showed that, in the absence of erythrocytes, the viability of lead-exposed HK-2 cells was not significantly different from the viability of unexposed HK-2 cells. A separate experiment demonstrated increased PS externalization in lead-exposed erythrocytes. And when lead-exposed erythrocytes were co-cultured with unexposed HK-2 cells, the HK-2 cells not only phagocytized the erythrocytes, but also showed increased production of reactive oxygen species, diminished viability, and greater expression of genes associated with kidney damage.^1^

Next, the *in vitro* findings were tested in rats. Samples of blood, spleen, and kidney tissue were collected from rats exposed to 0 or 1,000 ppm lead in drinking water for 12 weeks, then used for biochemical and histological analyses. These analyses revealed statistically significant changes consistent with kidney damage. A second 12-week experiment included tissue samples from rats that received 0, 250, or 1,000 ppm of lead acetate in drinking water; these results also supported the *in vitro* findings. The *in vivo* results also reflected two types of nephrotoxicity seen in epidemiological studies: proximal tubular nephropathy and interstitial fibrosis.^1^

William McClellan, a professor of medicine at Emory University in Atlanta who was not involved in the study, cautions against immediately extrapolating the findings to human health. He also notes that the findings will need replication. McClellan says, “I have a feeling that if they come up with comparable findings [for confirmation], there will be some strong interest in doing some human studies.”

## References

[1] Kwon SY Erythrophagocytosis of lead-exposed erythrocytes by renal tubular cells: possible role in lead-induced nephrotoxicity. Environ Health Perspect 123 2 120 127 2015; 10.1289/ehp.1408094 25302504 PMC4314246

[2] NTP. NTP Monograph: Health Effects of Low-Level Lead. Research Triangle Park, NC:National Toxicology Program, National Institute of Environmental Health Sciences, National Institutes of Health (June 2012). Available: http://ntp.niehs.nih.gov/ntp/ohat/lead/final/monographhealtheffectslowlevellead_newissn_508.pdf [accessed 14 January 2015]

[3] Soderland P Chronic kidney disease associated with environmental toxins and exposures. Adv Chronic Kidney Dis 17 3 254 264 2010; 10.1053/j.ackd.2010.03.011 20439094

[4] Jang WH Low level of lead can induce phosphatidylserine exposure and erythrophagocytosis: a new mechanism underlying lead-associated anemia. Toxicol Sci 122 1 177 184 2011; 10.1093/toxsci/kfr079 21482638

[5] HK-2 cells are a type of human proximal tubular epithelial cell.

